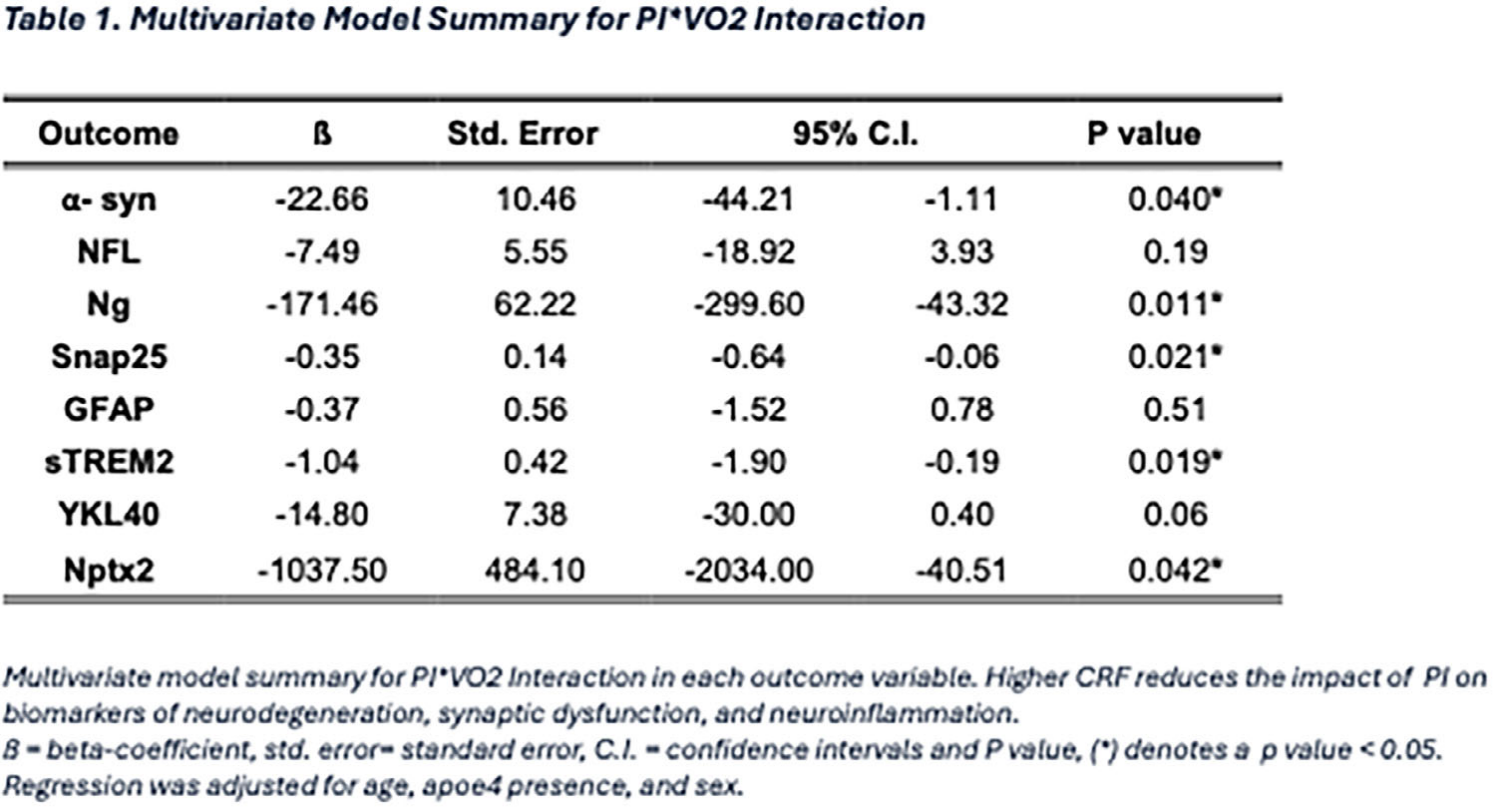# Cardiorespiratory Fitness Modifies the Association of Cerebral Pulsatility on CSF Biomarkers Related to Neurodegeneration, Synaptic Dysfunction, and Neuroinflammation

**DOI:** 10.1002/alz.089505

**Published:** 2025-01-09

**Authors:** Talia L. Brach, Brianne M. Breidenbach, Matthew P Glittenberg, Leah E Symanski, Tarun Naren, Ira Driscoll, Sarah R Lose, Catherine L. Gallagher, Sterling C. Johnson, Sanjay Asthana, Tobey J. Betthauser, Leonardo A. Rivera‐Rivera, Bruce P Hermann, Mark A. Sager, Kaj Blennow, Henrik Zetterberg, Cynthia M. Carlsson, Gwendlyn Kollmorgen, Clara Quijano‐Rubio, Dane B. Cook, Oliver Wieben, Ozioma C Okonkwo

**Affiliations:** ^1^ Wisconsin Alzheimer's Institute, Madison, WI USA; ^2^ Wisconsin Alzheimer's Disease Research Center, School of Medicine and Public Health, University of Wisconsin‐Madison, Madison, WI USA; ^3^ University of Wisconsin‐Madison, School of Medicine and Public Health, Madison, WI USA; ^4^ Geriatric Research Education and Clinical Center William S. Middleton VA Hospital, Madison, WI USA; ^5^ Wisconsin Alzheimer's Disease Research Center, Madison, WI USA; ^6^ Wisconsin Alzheimer’s Institute, University of Wisconsin‐Madison School of Medicine and Public Health, Madison, WI USA; ^7^ Clinical Neurochemistry Laboratory Sahlgrenska University Hospital, Mölndal Sweden; ^8^ Paris Brain Institute, ICM, Pitié‐Salpêtrière Hospital, Sorbonne University, Paris France; ^9^ Neurodegenerative Disorder Research Center, Institute on Aging and Brain Disorders, University of Science and Technology of China and First Affiliated Hospital of USTC, Heifei China; ^10^ Institute of Neuroscience and Physiology Sahlgrenska Academy at the University of Gothenburg, Gothenburg Sweden; ^11^ Wisconsin Alzheimer’s Disease Research Center, University of Wisconsin School of Medicine and Public Health, Madison, WI USA; ^12^ Department of Psychiatry and Neurochemistry, Institute of Neuroscience and Physiology, The Sahlgrenska Academy, University of Gothenburg, Mölndal, Gothenburg Sweden; ^13^ Hong Kong Center for Neurodegenerative Diseases, Hong Kong China; ^14^ UCL Institute of Neurology Queen Square London UK, London United Kingdom; ^15^ Roche Diagnostics GmbH, Penzberg Germany; ^16^ UK Dementia Research Institute at UCL, London United Kingdom; ^17^ Department of Psychiatry and Neurochemistry, Institute of Neuroscience and Physiology, The Sahlgrenska Academy at the University of Gothenburg, Mölndal Sweden; ^18^ Wisconsin Alzheimer's Disease Research Center, University of Wisconsin School of Medicine and Public Health, Madison, WI USA; ^19^ Roche Diagnostics International Ltd., Rotkreuz Switzerland; ^20^ University of Wisconsin School of Education, Madison, WI USA

## Abstract

**Background:**

Cerebral pulsatility (PI) is reportedly higher in individuals with AD and MCI compared to age matched controls and has been associated with greater beta‐amyloid (Aß) burden, but its relationship to other neurodegenerative biomarkers is unknown. Higher cardiorespiratory fitness (CRF) positively affects vascular function and is associated with lower PI in several large cerebral vessels. The relationship between PI, CRF, and biomarkers for neurodegeneration have not yet been characterized. Our objective was to examine a potential CRF modification of the relationship between PI and cerebrospinal fluid (CSF) biomarkers of neurodegeneration, synaptic dysfunction, and neuroinflammation.

**Methods:**

Cognitively unimpaired adults (n=32, Mean Age=64) from the Wisconsin Registry for Alzheimer's Prevention and the Wisconsin Alzheimer's Disease Research Center who met selection criteria were included. PI from bilateral MCA vessels were measured using 4D flow MRI and averaged (PI‐MCA_AVG_). CRF was measured as peak VO_2PEAK_ during a graded exercise treadmill test. CSF was obtained via lumbar puncture and measured using the NeuroToolKit, a panel of robust prototype assays (Roche Diagnostics International Ltd). The toolkit included measures of: neurodegeneration [α‐synuclein (α‐syn) and neurofilament light polypeptide (NfL)], synaptic dysfunction [neurogranin (Ng), synaptosomal‐associated protein 5 (SNAP‐25), and neuronal activity related protein 2 (nptx2)], and neuroinflammation [glial fibrillary acidic protein (GFAP), soluble triggering receptor expressed in myeloid cells (sTREM2), and chitinase‐3‐like protein 1 (YKL‐40)]. A single multivariate regression model, adjusted for sex, age, and APOE4, examined whether the association between PI‐MCA_AVG_ and CSF biomarkers varied as a function of CRF.

**Results:**

There was a significant interaction between PI and VO_2PEAK_ on α‐syn (ß = ‐22.66, p=0.04), Ng (ß =‐171.46, p=0.01), SNAP‐25 (ß = ‐0.35, p= 0.02), STREM2 (ß =‐1.04, p=0.02), and nptx2 (ß =‐1037, p=0.04) indicating more favorable profiles of CSF biomarkers with increasing VO_2PEAK_ despite elevated PI. No significant interactions were found for NfL, GFAP, or YKL40 (all p’s > 0.05).

**Conclusion:**

Our findings support the hypothesis that increased CRF influences the relationship between cerebral PI and CSF biomarkers related to neurodegeneration, synaptic dysfunction, and neuroinflammation indicating that improved CRF may offer protection against cerebrovascular alterations known to coincide with biomolecular changes in various types of dementia.